# Improving Technology to Diagnose Tuberculous Meningitis: Are We There Yet?

**DOI:** 10.3389/fneur.2022.892224

**Published:** 2022-05-30

**Authors:** Kenneth Ssebambulidde, Jane Gakuru, Jayne Ellis, Fiona V. Cresswell, Nathan C. Bahr

**Affiliations:** ^1^Infectious Diseases Institute, College of Health Sciences, Makerere University, Kampala, Uganda; ^2^Clinical Research Department, London School of Hygiene and Tropical Medicine, London, United Kingdom; ^3^Global Health and Infection, Brighton and Sussex Medicine School, Brighton, United Kingdom; ^4^Division of Infectious Diseases, Department of Medicine, University of Kansas Medical Center, Kansas City, KS, United States

**Keywords:** tuberculosis, TB meningitis, tuberculous meningitis, diagnostic testing, molecular testing, cerebrospinal fluid

## Abstract

Diagnosis of tuberculous meningitis (TBM) remains challenging due to a paucity of high-performance diagnostics. Even those that have reasonable sensitivity are not adequate to ‘rule out' TBM. Therefore, a combination of clinical factors alongside microbiological, molecular, and radiological investigations are utilized, depending on availability. A low threshold for starting empiric therapy in the appropriate clinical scenario remains crucial for good outcomes in many cases. Herein, we review the current TBM diagnostics landscape with a focus on limitations frequently encountered, such as diagnostic test performance, cost, laboratory infrastructure, and clinical expertise. Though molecular technologies, particularly GeneXpert MTB/Rif Ultra, have been a step forward, diagnosis of TBM remains difficult. We also provide an overview of promising technologies, such as cerebrospinal fluid (CSF) lactate, a new lipoarabinomannan test (FujiLAM), metagenomic next-generation sequencing, and transcriptomics that may further improve our TBM diagnostic capacity and lead to better outcomes.

## Introduction

Tuberculous meningitis (TBM) is the deadliest form of tuberculosis (TB). In 2019, an estimated 164,000 cases occurred globally, of whom 23% were in people living with HIV (PLHIV) (1). Mortality is 10–24% in HIV-negative individuals, and 46–67% in PLHIV (2, 3). Even among survivors, long-term neurologic disability occurs in 22–43% (2). TBM outcomes are poor, in part, due to the delayed or missed detection of *Mycobacteria tuberculosis* in cerebrospinal fluid (CSF).

A comprehensive history, physical examination, and a combination of radiological, microbiological, and molecular tests are frequently necessary to diagnose TBM, and even with all this information in hand, there is often diagnostic uncertainty. Individuals with TBM may present with headaches, fever, neck stiffness, seizures, visual changes, and behavioral and/or cognitive disturbances with a mean duration of 12 days (4–6). Physical examination may reveal nuchal rigidity, focal neurological deficits, and altered consciousness on the Glasgow Coma Scale (4, 5, 7, 8). While these signs and symptoms are suggestive of meningitis, they are not specific to TBM.

**Figure 1:**
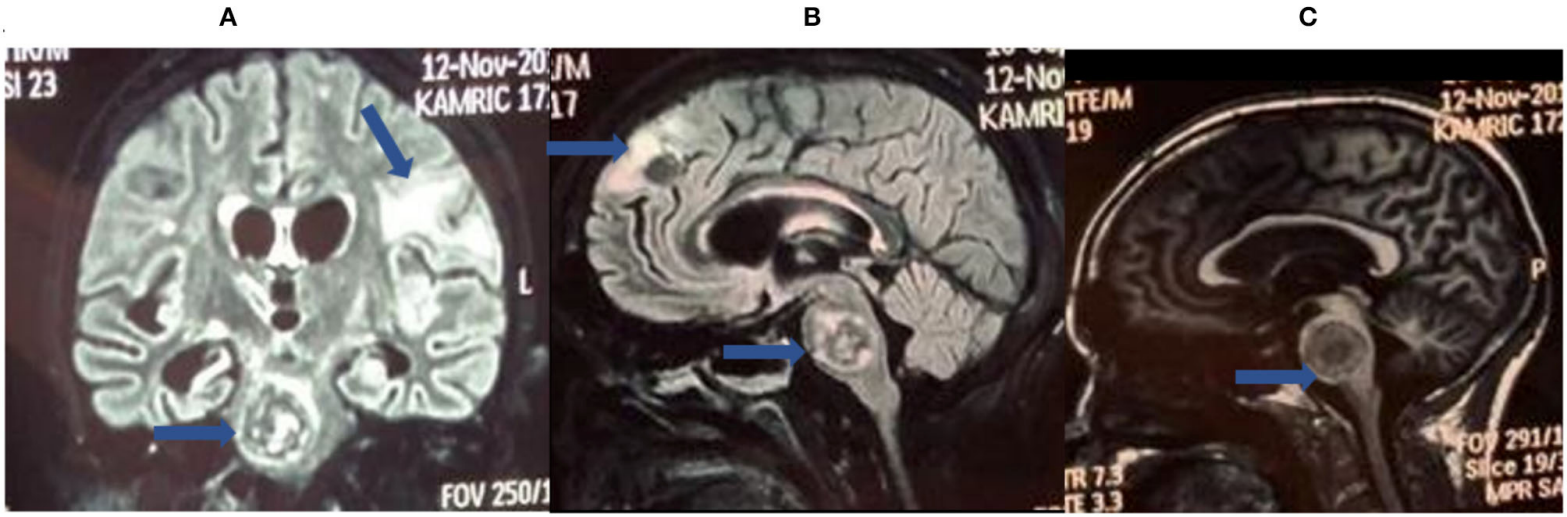
Brain magnetic resonance imaging (MRI) for tuberculous meningitis (TBM) diagnosis. These are selected images from a brain MRI obtained from a patient with definite TBM showing cerebral space occupying lesions (tuberculomas) in the brainstem **(A–C)** and frontal lobe along with white matter ischemic changes **(B)**.

Brain imaging (meningeal enhancement, parenchymal mass lesions (tuberculomas), infarctions, and/or hydrocephalus) (Figure 1) and basic CSF testing are often not specific for TBM (9–11).

Imaging is not routinely available in many resource-limited areas. Microbiological and molecular tests are more specific but are not sensitive enough to detect all cases of TBM. Early, empiric treatment remains important in many cases. In this review, we discuss the current state of TBM diagnostics and promising technologies that may further improve our ability to rapidly detect TBM and lead to better outcomes.

## Research Case Definitions

Due to the complexity in making a TBM diagnosis, standardized criteria have been formulated, designed to allow comparison of research studies (12). These standardized diagnostic criteria utilize clinical parameters alongside laboratory and radiological investigations (if available) but were not designed for clinical use (Table 1). The consensus case definitions can be used for any population and categorize patients into the following sub-groups: definite TBM, probable TBM, possible TBM, or not TBM based on a score of 0–20. Individuals with definite TBM have microbiological and/or molecular evidence of *M. tuberculosis* [acid-fast bacilli (AFB) smear, culture, or commercially available polymerase chain reaction (PCR)] in their CSF or on autopsy. Probable TBM cases have a clinical syndrome consistent with TBM and a diagnostic score of >10 (without brain imaging) or >12 (with brain imaging) and no alternative diagnosis. Individuals with possible TBM have a diagnostic score of 6–9 (with no brain imaging) or 9–11 (with brain imaging) and no alternative diagnosis. Individuals in the not TBM category have <6 points or an alternative diagnosis. Clinical criteria have also been developed but none have been adequately validated across cohorts such that they are reliable for routine use (13).

**Table 1:** Uniform case definition (12).

	**Diagnostic score**
**A. Clinical criteria**	**(Maximum category score** **=** **6)**
Symptom duration of more than 5 days	4
Systemic symptoms suggestive of tuberculosis (1 or more of the following): weight loss (or poor weight gain in children), night sweats, or persistent cough for more than 2 weeks	2
History of recent (within past year) close contact with an individual with pulmonary tuberculosis or a positive TST or IGRA (only in children <10 years of age)	2
Focal neurological deficit (excluding cranial nerve palsies)	2
Cranial nerve palsy	1
Altered consciousness	1
**B. CSF criteria**	**(Maximum category score** **=** **4)**
CSF appearance	1
Cells: 10–500 per uL	1
Lymphocyte predominance (>50%)	1
Protein concentration >1 g/L	1
CSF to plasma glucose ratio of <50% or an absolute CSF glucose concentration <2.2 mmol/L	1
**C. Cerebral Imaging criteria**	**(Maximum category score** **=** **6)**
Hydrocephalus	1
Basal meningeal enhancement	2
Tuberculoma	2
Infarct	1
Pre-contrast hyperdensity	2
**D. Evidence of tuberculosis elsewhere**	**(Maximum category score** **=** **4)**
Chest radiograph suggestive of active tuberculosis: signs of tuberculosis =2, miliary tuberculosis =4	2/4
CT/MRI/ultrasound evidence of tuberculosis outside the CNS	2
AFB identified or Mycobacteria tuberculosis cultured from another source i.e., sputum, lymph node, gastric washing, urine, blood culture	4
Positive commercial M. tuberculosis NAAT from extra-neural specimen	4
**Total score** **=**	
**Definite TB meningitis**: Patients would fulfill criterion A or B: **A**. Clinical entry criteria plus one or more of the following: AFB seen in the CSF; Mycobacteria tuberculosis cultured from CSF; or a CSF positive commercial NAAT **B**. AFB seen in the context of histological changes consistent with tuberculosis in the brain or spinal cord with suggestive symptoms or signs and CSF changes or visible meningitis on autopsy.	
**Probable TB meningitis**- total diagnostic score ≥12 (imaging available) or ≥10 (imaging unavailable)	
**Possible TB meningitis**- total diagnostic score 6–11 (imagine available) or 6–9 (imaging unavailable)	
**Not TB meningitis**- total diagnostic score <6 or alternative cause identified	
**Exclusion of alternative diagnoses:** An alternative diagnosis must be confirmed microbiologically (by stain, culture, or NAAT when appropriate), serologically (e.g., syphilis), or histopathologically (e.g., lymphoma). The list of alternative diagnoses that should be considered, dependent upon age, immune status, and geographical region include: pyogenic bacterial meningitis, cryptococcal meningitis, syphilitic meningitis, viral meningo-encephalitis, cerebral malaria, parasitic or eosionophilic meningitis (Angiostrongylus cantonesis, Gnathostoma spinigerum, toxocariasis, cysticercosis), cerebral toxoplasmosis and bacterial brain abscess (space-occupying lesion on cerebral imaging) and malignancy (e.g., lymphoma)	

*TST, tuberculosis skin test; IGRA, interferon-gamma release assay; NAAT, nucleic acid amplification test; AFB, acid fast bacilli; CSF, cerebrospinal fluid; CNS, central nervous system; CT, computed tomography; MRI, magnetic resonance imaging*.

## Basic Csf Tests

Persons with TBM generally have CSF lymphocyte-predominant pleocytosis (CSF white blood cell (WBC) count: 50–1,000 cells/μl), increased CSF protein > 45 mg/dl, and CSF: blood glucose ratio <0.5 or CSF glucose below 2.2 mmol/L. However, this is not always the case, especially among PLHIV where CSF WBC counts may be normal or minimally increased (4, 14–16). Additionally, the white cell differential may show neutrophil predominance (particularly in early disease), which has been associated with immune reconstitution inflammatory syndrome (17–20). Of these CSF parameters, CSF glucose below 2.2 mmol/L or a CSF:plasma ratio below 0.5 best predicts bacteriologically confirmed TBM (21, 22). None of these tests are specific for TBM.

Given that delay in initiating treatment leads to poor prognosis, empirical treatment is often initiated based on the non-specific CSF parameters noted above. However, empiric treatment, while life-saving for those with TBM, also exposes many (who ultimately do not have TBM) to unnecessary toxicities from tuberculosis treatment. Thus, identification of *M. tuberculosis* is paramount to make a definitive TBM diagnosis, ideally identifying those with true TBM rapidly and avoiding unnecessary treatment in those without TBM.

## Traditional Mycobacteriological Diagnostics

Ziehl–Neelsen (ZN) staining of CSF for AFB is a cheap, rapid, and widely available method for diagnosing TBM. Our recent meta-analysis of 2,450 individuals with suspected TBM found a pooled sensitivity of CSF AFB smear of 9% (95% *CI*: 3–22%) and a pooled specificity of 100% (95% *CI*: 90–100%) compared to the reference standard of CSF culture or NAAT or both (23). With ~10,000 organisms required to obtain a positive smear, TBM (frequently paucibacillary) is often missed by this technique (24) (Table 2). Sensitivity depends on the skills of the microscopist, the volume of CSF used (a proxy for the microbiological load), and the time spent in analyzing the specimen. In one study, CSF volume > 6 ml and microscopist time analyzing CSF for AFB > 20 min had a sensitivity of 52% compared with case definitions (25). Another approach involves intracellular AFB staining—a single-center study reported 100% sensitivity vs. 27.6% for conventional ZN staining using this technique (26). Yet, compared with the consensus case definitions, this modification yielded a sensitivity of 34.5%, similar to the conventional AFB smear (33.9%) in a large (*N* = 618) prospective, multicenter study (22). In general, efforts to duplicate promising modifications to the ZN smear (e.g., technique, volume, or time-based) have proven difficult and AFB smear remains low yield in most settings.

**Table 2:** Diagnostic performance for selected tests for tuberculous meningitis (TBM).

**Test**	**Limit of detection (CFU/mL)**	**Sensitivity %(95%CI)**	**Specificity %(95%CI)**	**Reference standard**	Limitations^*^	**References**
Culture	<10 CFU (Liquid media) 10–100 (Solid media)	50–70	100	Consensus case definitions	Cost, infrastructure, TAT, sensitivity	(27, 28)
AFB smear	10,000	10–20	100	Culture	Sensitivity, user variability	(24, 25)
Xpert	~110	71.1 (62.8–79.1)	96.9 (95.4–98)	Culture	Cost, infrastructure, sensitivity	(29, 30)
Xpert Ultra	~10–15	89.4 (79.1–95.6)	91.2 (83.2–95.7)	Culture	Cost, infrastructure	(29, 30)
CSF Alere LAM	Not available	22–33	94–96	Xpert Ultra or culture	Sensitivity	(31, 32)
CSF FujiLAM	Not available	74 (56–87)	91 (82–97)	Consensus case definitions	Replication of study, possibly cost, not commercially available	(33)

*AFB, Acid fast bacilli; CSF, cerebrospinal fluid; LAM, lipoarabinomannan; TBM, tuberculous meningitis; CFU, colony forming units; 95% CI, 95% confidence intervals; TAT, turnaround time.*

^*^None of the tests have adequate negative predictive value to “rule-out” TB meningitis.

A CSF culture for *M. tuberculosis* has a better sensitivity than AFB smear for TBM (25, 34). Compared with case definitions, solid Löwenstein Jensen (LJ) culture has 50–70% sensitivity with a mean time to positivity of 3–5 weeks whereas liquid mycobacteria growth indicator tube (MGIT) culture has similar to slightly higher sensitivity with results in as short as 2 weeks (25, 35). MGIT has a limit of detection (LOD) of ~10 colony forming units (CFU)/ml, and LJ culture's LOD is 10–100 CFU/mL (36). Centrifuging a large volume of CSF may improve culture sensitivity, similar to AFB smear (25). Despite these potential strategies to improve test performance, CSF culture results are usually too slow for clinical decision-making. Additionally, *M. tuberculosis* culture requires a biosafety level 3 laboratory and is relatively expensive at $13–50 per unit. However, when positive, culture is important for drug susceptibility testing.

## Molecular MTB Diagnostics

Nucleic acid amplification tests (NAATs), such as PCR, have been of particular interest for improving TBM diagnosis. GeneXpert MTB/Rif (Xpert, Cepheid, Sunnyvale, CA, USA) is a cartridge-based, fully automated, rapid (<2 h) PCR test that also detects rifampicin resistance. Xpert is now widely distributed in resource-limited settings albeit underutilized and with concentrations at reference centers (37, 38). In a recent Cochrane review (2021), CSF Xpert had a pooled sensitivity of 71.1% (95% *CI*: 62.8–79.1%) and a pooled specificity of 96.9% (95% *CI*: 95.4–98.0%) against CSF culture (29). Xpert sensitivity is also improved when more than 6 ml of CSF are centrifuged before testing (39, 40). In 2013, Xpert was recommended by the WHO as the preferred first test for TBM (41). Since then, Xpert is a possible contributor to a decline in in-hospital mortality from 57 to 41% in a Ugandan cohort of HIV/TBM co-infected individuals (42). Yet, even with these advantages, Xpert has inadequate negative predictive value (NPV) to ‘rule out' TBM and cannot be used as a single test with that intent (43). Cost and laboratory infrastructure requirements are also limitations.

Due to this limitation (and concerns about reliability of rifampin resistance detection), Xpert was re-engineered with technical enhancements (a larger specimen volume reaching the PCR reaction, additional probes for two other DNA targets, optimized microfluidics, and PCR cycling) and an additional ‘trace' category for the lowest bacillary load detected as GeneXpert MTB/Rif Ultra (Xpert Ultra) has improved sensitivity and more reliable detection of rifampicin resistance. Xpert Ultra has a lower LOD (15.6 CFU/mL) compared with 112.6 CFU/ml for Xpert (30). Practically, this means that Xpert Ultra can yield a positive result with fewer bacilli. Against CSF culture, Xpert Ultra has a pooled sensitivity of 89.4% (95% CI: 79.1–95.6%) and a pooled specificity of 91.2% (95% CI: 83.2–95.7%) (29). The WHO, in 2017 recommended the adoption of Xpert Ultra in diagnosing TBM to replace Xpert as the first line test (44, 45). Importantly, Xpert Ultra also may detect *M. tubrerculosis* in cases missed by culture as well (46). Unfortunately, Xpert Ultra also has inadequate NPV to ‘rule-out' TBM, varying from 61·1% (49·6–71·5%) in Vietnam to 92.7% (87.6–96.2%) in Uganda, such that it still cannot be used as the only test to exclude TBM (47–49). Cost and laboratory infrastructure requirements are limitations here as well.

## Promising Tests

Lipoarabinomannan (LAM) is a phosphorylated lipopolysaccharide in the *Mycobacteria* cell wall (50). LAM can be detected in the urine of those with disseminated TB. Currently, there are two point-of-care tests to detect LAM. Alere TB-LAM (Alere Determine TB-LAM, Abbott, Chicago, USA) is commercially available and uses conventional lateral flow immunoassay technology and polyclonal antibodies. FujiLAM (Fujifilm SILVAMP TB-LAM, Fujifilm, Japan) combines a pair of high-affinity monoclonal antibodies for *Mycobacterium tuberculosis*-specific lipoarabinomannan epitopes and a silver-amplification step but is not yet commercially available. Both tests perform better in urine among PLHIV with more advanced immune suppression (CD4 <100 cells/μl) given the higher mycobacterial burden in that population (51, 52).

In a prospective study of PLHIV in Uganda, CSF Alere TB-LAM had a sensitivity of 33% (95% *CI*: 9.9–65.1%) and a specificity of 96% (95% *CI*: 85.5–99.5%) vs. probable or definite TBM diagnosed by Xpert Ultra (31). In another cohort made up predominantly of PLHIV in Zambia, CSF Alere LAM had a sensitivity of 21.9% and a specificity of 94.2% compared with CSF culture (32). There has been one published study of FujiLAM on CSF for TBM diagnosis to date: among 101 Ugandans, primarily PLHIV, FujiLAM had a sensitivity of 52% and a specificity of 98% (87% *CI*: 87–100%) against probable/definite TBM and a sensitivity of 74% (95% *CI*: 56–87%) against definite TBM (33). These point-of-care tests have considerable advantages over Xpert Ultra or culture in that they are rapid (30 min) and do not require significant laboratory infrastructure (the major advantage vs. Xpert Ultra). Their best use may end up being in combination with Xpert Ultra, but that is to be determined as additional studies are urgently needed. Early results certainly suggest higher sensitivity with FujiLAM than Alere LAM, but that must be confirmed and the former is not yet commercially available.

Lastly, CSF lactate, which can be performed at the bedside with a handheld device to aid prompt treatment decisions, may be utilized in support of TBM diagnosis. In a prospective study of 575 Ugandan adults with suspected meningitis, bedside CSF lactate was higher in patients with definite TBM [8.1 mmol/l, interquartile range (IQR) 6.5–9.8] than probable TBM (4.0 mmol/l, IQR 2.5–9.5), possible TBM (2.6 mmol/l, IQR 2.1, 3.9), and not-TBM (3.6 mmol/l, IQR 2.6–5.1). At a cut point of >5.5 mmol/L, CSF lactate was able to diagnose definite/probable TBM with a sensitivity of 67.7% (similar to Xpert Ultra), a specificity of 80.3%, and a negative predictive value of 95.4% (*In Press* at Microbiology Spectrum). The addition of CSF lactate as a ‘rule-in' test to the basic CSF tests (total protein, glucose, and WBC count) might improve their diagnostic performance but more studies are needed as other central nervous system (CNS) diseases, such as bacterial and cryptococcal meningitis (53, 54), cerebral malaria (55), CNS lymphoma (56), seizure disorders (57), and coronavirus disease 2019 (COVID-19) with neurological symptoms (58) can also cause CSF lactate elevation.

## Tests Not In Routine Use

Most of the current tests in clinical use are based on detecting the *M. tuberculosis* bacilli (e.g., microscopy and culture) or its components (e.g., LAM and Xpert Ultra). However, the host-immune response to *M. tuberculosis* has also been of interest for diagnosis. The interleukin-12-interferon gamma (IFNg) axis is required to control *M. tuberculosis* (an intracellular pathogen) (59, 60). IFNg release assays (IGRAs) were designed to measure the IFNg-secreting T lymphocytes by enzyme-linked immunospot assay (ELISpot) (TSPOT.TB, Oxford Immunotec, Oxford, UK) or measure the concentration of IFNg by enzyme-linked immunosorbent assay (ELISA) (QuantiFERON-TB Gold In-Tube, Cellestis, Carnegie, Australia) (61). In a recent meta-analysis, as a TBM diagnostic, CSF IGRA had a sensitivity of 77% (95% *CI*: 69–84%) and a specificity of 88% (95% *CI*: 74–95%) (62). However, major limitations, such as cost, infrastructure requirements, and the tests' inability to separate latent from active TB infection have limited their utility for TBM.

Measuring CSF adenosine deaminase (ADA) has also been studied as a diagnostic test for TBM. ADA is a universal enzyme involved in purine metabolism in all human cells whose deficiency primarily affects lymphocyte function (63). In TBM, ADA CSF levels are increased (64). As a TBM diagnostic test, CSF ADA had a pooled sensitivity of 89% (95% *CI*: 84–92%) and a specificity of 91% (95% *CI*: 87–93%) in a recent systematic review and meta-analysis (65). However, the clinical use of CSF ADA has been minimal due to its high cost, laboratory infrastructure, and personnel requirements, relatively poor specificity, as well as significant heterogeneity in study results and design.

Antibody tests have been developed for certain *Mycobacteria* antigens (66, 67). In a recent review of various CSF antibody tests, the pooled sensitivity of anti-M37Ra from five studies was 91% (95% *CI*: 71–98%), anti-antigen 5 from eight studies was 84% (95% *CI*: 71–92%), and anti-M37Rv from 12 studies was 84% (95% *CI*: 71–92%) (68). However, variability in reference standards makes these studies difficult to compare. *M. tuberculosis* antibody tests are not currently recommended for use in clinical practice.

Other molecular tests with potential for TBM but requiring additional evaluation include loop-mediated isothermal amplification (LAMP), and Truenat MTB Plus (Truenat, Molbio Diagnostics, Verna, India). LAMP is a PCR-based test with a sensitivity of 88–96% and a specificity of 80–100% against culture-confirmed TBM but is highly dependent on primer design and requires further study (69, 70). Truenat had a sensitivity of 85.5 vs. 96% for Xpert Ultra in one study using definite TBM as a reference standard, these results require replication (71).

## Future Directions

Kwon et al. evaluated the diagnostic performance of CSF cytokines/chemokines in diagnosing TBM in the CSF from 10 individuals with TBM (two with definite TBM and eight with probable TBM) and 45 individuals with no TBM (72). They observed the following sensitivities and specificities of CSF cytokine/chemokines: CSF Interleukin (IL)-12p40 > 52.04 pg/ml, 80.0% (95% *CI*: 44.4–97.5%) and 73.3% (95% *CI*: 58.1–85.4%); CSF IL-13 > 0.44 pg/ml, 90.0% (95% *CI*: 55.5–99.8%) and 46.7 (95% *CI*: 31.7–62.1%); and CSF CCL3/macrophage inflammatory protein (MIP)-1α >8.83 pg/ml, 80.0% (95% *CI*: 44.4–97.5%) and 62.2% (95% *CI*: 46.5–76.2%), respectively. None of these tests would have the specificity to be used as a singular diagnostic test for TBM. Based on these data, it is unclear whether these tests could play a role as part of a multi-test approach. Further studies on CSF cytokines and chemokines are warranted, particularly in combination with other diagnostic modalities.

Metagenomic next-generation sequencing (mNGS) analyzes all microbial genomes in a clinical sample in an unbiased fashion. In a retrospective analysis of 51 inpatients without HIV suspected TBM, CSF mNGS had a sensitivity of 84.4% (38/45, 95% *CI*: 69.9–93.0%) and a specificity of 100% (6/6, 95% *CI*: 51.68%−100%) against consensus case definitions (73). Using definite TBM as a reference standard in another cohort (*N* = 12), mNGS had a sensitivity of 66.67% and a specificity of 100% in 23 patients (74). In a recent large (*N* = 368) study made up of predominantly PLHIV, combining mNGS with machine learning had a sensitivity of 88.9% (95% *CI*: 51.8–99.7%) and a specificity of 86.7% (95% *CI*: 76.8–93.4%) (65/75) against definite and probable TBM (75). This combination was also able to detect 8 additional TBM cases that had initially been classified as possible TBM. However, cost, laboratory expertise and infrastructure requirements are currently major barriers to its implementation.

Clustered regularly interspaced short palindromic repeats (CRISPR)-associated proteins (Cas) have begun to be analyzed as tools for TBM diagnosis. This technology can detect low levels of *M. tuberculosis* when coupled with DNA amplification. Using this technology, Ai et al. found an LOD of 50 CFU/ml in sputum for individuals with pulmonary tuberculosis (76). Using clinical TBM (not the consensus research definitions) as a reference standard, CSF CRISPR-MTB had a sensitivity of 73% in a small study (*n* = 26). Although more sensitive than Xpert (54%) or culture (23%), this study is limited by its use of a non-standard reference, no controls, and small numbers of putative TBM (76). Further studies of this technology are warranted and, if promising, the technology would need significant adaptation to address real-world feasibility.

Further characterization of the host immune response *via* transcriptomic technologies is another expanding area of interest in TBM diagnostics. RNA sequencing has revealed differential expression of transcripts in TBM vs. healthy controls, and that transcripts differ by the site of CSF collection (77). Ventricular CSF shows significant transcripts associated with neuronal excitation and injury compared with the lumbar CSF profile that represents protein translation and cytokine signaling. Whether a CSF-based TBM host response assay could be developed (similar to the blood-based and Xpert MTB Host Response 3-gene assay) is uncertain. Further, transcriptomic technology may provide additional insights into the pathophysiology of TBM with the potential for the development of novel host-directed therapies and diagnostics.

## Conclusion

The diagnosis of TBM remains a challenge. However, recently developed tests, such as Xpert Ultra have improved the overall possibility of obtaining an accurate diagnosis in persons suspected to have TBM, especially when compared to traditional tests, such as AFB smear and culture. The quest for a bedside point of care test continues and FujiLAM may be a step in the right direction. With the ongoing advances in DNA/RNA sequencing technologies and machine learning, perhaps host-based diagnosis may become feasible in TBM. Despite the existence of uniform case definitions for over 10 years, they are not always utilized. We strongly encourage the use of the consensus case definitions of all studies of TBM diagnostic tests to improve comparisons between studies.

Equally as critical to developing highly accurate diagnostics for TBM is ensuring that they are implemented in TB endemic settings in such a way that they have the potential to change management and outcomes. For promising new diagnostics, generating robust evidence based on impact and cost-effectiveness is needed such that they can be evaluated by the WHO and then taken up in national policy and practice. We advocate for multi-center trials evaluating the diagnostic performance of TBM diagnostic tests. These diagnostic performance studies should ideally be conducted in hyper-endemic areas and include both adults and children, as well as individuals with HIV infection and those who are HIV negative.

For many of the tests described, limitations include the availability of the tests, costs, the need for laboratory infrastructure, and highly trained personnel. Ultimately, policymakers need to prioritize diagnostics for TBM, and researchers, in addition to providing information related to diagnostic accuracy, must consider cost-effectiveness as well. Empirical pre-treatment which is commonly done, might further compound the poor diagnostic performance of TBM tests. The journey from the laboratory bench to the bedside is a long one, but one that must be taken to find tests that can reduce death and disability from this devastating disease.

## Author Contributions

All authors listed have made a substantial, direct, and intellectual contribution to the work and approved it for publication.

## Funding

NB receives research support from the National Institute of Neurological Disorders and Stroke of the National Institutes of Health, award K23NS110470. JE is funded through a Wellcome Trust Clinical PhD Fellowship in Global Health Research. FC receives research funding through a Wellcome PhD fellowship (210772/Z/18/Z) and NIHR Academic Clinical Lectureship (CL-2020-27-001).

## Conflict of Interest

The authors declare that the research was conducted in the absence of any commercial or financial relationships that could be construed as a potential conflict of interest.

## Publisher's Note

All claims expressed in this article are solely those of the authors and do not necessarily represent those of their affiliated organizations, or those of the publisher, the editors and the reviewers. Any product that may be evaluated in this article, or claim that may be made by its manufacturer, is not guaranteed or endorsed by the publisher.
